# Postoperative rehabilitation and quality of life evaluation for transoral endoscopic thyroidectomy vestibular approach

**DOI:** 10.1038/s41598-024-65589-x

**Published:** 2024-06-26

**Authors:** Yu Mao, Shiwei Zhou, Peng Wu, Wu Li, Hui Li, Zhiyuan Wang, Xibin Xia, Xiaohua Song, Mingming Wang, Xiaowei Peng

**Affiliations:** 1 Department of Thyroid Surgery, The Affiliated Cancer Hospital of Xiangya School of Medicine, Central South University/Hunan Cancer Hospital, Changsha, 410013 Hunan Province People’s Republic of China; 2 Department of Thyroid Surgery, The Second Xiangya Hospital, Central South University, Changsha, 410011 Hunan People’s Republic of China; 3 Department of Medical Ultrasound, The Affiliated Cancer Hospital of Xiangya School of Medicine, Central South University/Hunan Cancer Hospital, Changsha, 410013 Hunan People’s Republic of China; 4 Department of Diagnostic Radiology, The Affiliated Cancer Hospital of Xiangya School of Medicine, Central South University/Hunan Cancer Hospital, Changsha, 410013 Hunan People’s Republic of China

**Keywords:** Transoral endoscopic thyroidectomy vestibular approach, Quality of life, Postoperative rehabilitation, Cervical range of motion, Questionnaire, Surgical oncology, Quality of life

## Abstract

There are no targeted rehabilitation training modalities and assessment tools for patients after transoral endoscopic thyroidectomy vestibular approach (TOETVA). Herein, we develop a new assessment questionnaire and rehabilitation training modality and evaluate its safety and effectiveness. The THYCA-QoL-TOETVA questionnaire was compiled, and reliability and validity analyses were performed. Patients were divided into the new rehabilitation training group (N) or the conventional rehabilitation training group (C), and 1:1 propensity score matching (PSM) was performed after administering questionnaires to patients in both groups. Cervical range of motion (CROM) data were also measured and collected for statistical analysis. The questionnaire used in this study showed good expert authority, coordination, internal consistency, and questionnaire reliability. A total of 476 patients were included after PSM, and the questionnaire results showed that recovery and quality of life were better in the N group than in the C group (124.55 ± 8.171 vs. 122.94 ± 8.366, *p* = 0.026). Analysis of cervical spine mobility showed that rehabilitation was better in the N group compared to the C group at postoperative one month (flexion: 1.762°, extension: 4.720°, left lateral bending: 3.912°, right lateral bending: 4.061°, left axial rotation: 5.180°, right axial rotation: 5.199°, *p* value all of these < 0.001), and at postoperative three months (flexion: 2.866°, extension: 2.904°, left lateral bending: 3.927°, right lateral bending: 3.330°, left axial rotation: 4.395°, right axial rotation: 3.992°, *p* value all of these < 0.001). The THYCA-QoL-TOETVA provides an appropriate and effective tool for measuring the postoperative quality of life of TOETVA patients. This new rehabilitation training can effectively alleviate the problem of limited neck movement and improve the quality of life of patients after TOETVA surgery.

**Trial registration**: ChiCTR2300069097.

## Introduction

In recent years, the Transoral Endoscopic Thyroidectomy Vestibular Approach (TOETVA) surgery has garnered increasing attention and adoption owing to its minimally invasive and scarless attributes^1–3^. Nevertheless, there remains a relative scarcity of research concerning the quality of life and rehabilitation of patients post-TOETVA.

The role of postoperative rehabilitation exercises is pivotal in the comprehensive recovery of thyroid cancer patients. The therapeutic phase, when complemented by targeted, rational, and effective rehabilitation, can markedly improve the patient’s quality of life (QOL)^4^. Present rehabilitation programs and QOL assessment questionnaires predominantly focus on conventional open thyroid surgery, inadequately addressing the distinct anatomical considerations of TOETVA^5,6^. In contrast to conventional procedures, TOETVA involves the creation of a surgical tunnel in the chin region, resulting in damage to muscles and soft tissues, leading to postoperative chin and lip swelling and thereby impacting the postoperative QOL. Consequently, meeting the specific needs of TOETVA patients necessitates the development of specialized QOL assessment questionnaires for an accurate reflection of their postoperative well-being. Additionally, tailored rehabilitation programs are imperative to effectively address the unique challenges encountered by these patients during the postoperative period.

This study endeavors to formulate a postoperative rehabilitation method and a QOL questionnaire meticulously designed to cater to the distinctive features of TOETVA. Furthermore, the evaluation will encompass the safety of the exercise method and the validity of the questionnaire.

## Methods

### Study cohort

This was a retrospective cohort study conducted at a tertiary academic health care institution. Seven hundred and thirty-three patients with differentiated thyroid cancer who underwent TOETVA from January 2020 to September 2022 while admitted to Hunan Cancer Hospital were selected as the study population and divided into new rehabilitation training group (N group, 263) and control group (C group, 470) according to different postoperative rehabilitation training modalities. The experimental group used new rehabilitation training (N group), while the control group used conventional rehabilitation training (C group). All surgical and postoperative rehabilitation instruction was done by the same surgeon at Hunan Provincial Cancer Hospital in Changsha, China. The study was approved by the Medical Ethics Committee of Hunan Cancer Hospital and the subjects gave their informed consent for participation. The study was registered with the Chinese Clinical Trials Registry under registration number ChiCTR2300069097, in accordance with the World Medical Association Declaration of Helsinki 2013. This study was compliant with the STROBE guidelines^7^.

### Inclusion and exclusion criteria

#### Inclusion criteria

(1) 18 years of age or older; (2) Patients diagnosed with differentiated thyroid cancer; (3) Underwent TOETVA; (4) No other postoperative complications; (5) Understood the content of this study and signed an informed consent form.

#### Exclusion criteria

(1) Endoscopic surgery was converted to open technique; (2) Patient’s previous history of neck surgery; (3) Patient’s previous history of cervical spine disease; (4) Patient’s missed visits or insisted on withdrawal from the trial.

### Rehabilitation training

All patients enrolled in the group received preoperative rehabilitation education, practical bedside instruction after surgery, and were urged to follow the study rehabilitation instruction video to continue rehabilitation training after discharge.

#### Experimental group

The rehabilitation included active training and passive rehabilitation (Video 1), mainly including:

##### Anterior cervical fascia release

(1) Push and press vertically up and down 1 cm from the tip of the chin along the lower edge of the mandible to the angle of the mandible; (2) Push and press vertically up and down 1 cm from the head of clavicle along the upper and lower edge of the clavicle to the acromion; (3) Start from the midpoint of the clavicle with the second knuckle of the back of the four fingers and push and rub the skin and subcutaneous fascia of the anterior neck from the bottom to the lower edge of the mandible, paying attention to keep the contact and pressure between the fingers and the skin in the middle, five sets in total.

##### Superficial fascial release of neck and shoulder

(1) Thumb or index finger with the finger belly (the soft part of the palm by the last digit) from the mastoid behind the ear to about two horizontal fingers in the direction of the occipital ridge, vertical up and down pushing and pressing 1 cm; (2) Flex the patient’s head to the lateral front, hands with the second knuckle of the back of the four fingers from the shoulder peak and scapular gonad, from bottom to top pushing and rubbing the skin and subcutaneous fascia of the neck and shoulder to the mastoid behind the ear to about two horizontal fingers along the occipital ridge backwards, pay attention to always maintain the contact and pressure between the finger belly and the skin in the middle, do not interrupt. Repeat step two five times in total.

##### Passive stretching of the neck

The operator holds the patient’s back with one hand, presses the patient’s forehead with the other, and stretches the patient’s head backward as far as possible until pain is produced, for 30 s and then rests for 30 s, for a total of five sets.

##### Self-stretching exercises for the neck

Sitting, place one hand under the hip or pull the lower edge of the chair to fix the shoulder locked in place, with the other hand apply palm pressure to pull the head to the other side, for 30 s, then use the hand to assist the lower front flexion, for 30 s, and finally rotate to the outside, for 30 s, rest for one minute between sets, five sets in total.

##### Neck stretching exercise

Stretch the head and neck backward as far as possible, pay attention to open the mouth during the stretching process to ensure the maximum stretching amplitude, keep the head position unchanged until the end, repeat the mouth action three times, 10 times/group, three groups in total.

Rehabilitation exercises are recommended to start 10 days post-surgery, with patients performing the exercises three times a day. The recovery was evaluated at the one-month postoperative follow-up, and the frequency of exercise was adjusted to 1–2 times per day, which continued to the three-month follow-up.

#### Control group^8^

1. Relax your shoulders and neck sufficiently.
2. Turn face to the left and right, amplitude < 60°; look up and down, amplitude < 30°, alternately, 5–10 min at a time, three times a day.
3. 5–10 days after surgery, functional exercises of the shoulder and neck were performed. Including turn shoulders round and round and raise hands fully then lower them and upper arm wall climbing, 5–10 min at a time, three times a day.

### Questionnaire development

#### Source of questionnaire entries

The THYCA-QoL-TOETVA (Thyroid Cancer-Specific Health-Related Quality of Life Questionnaire for Transoral Endoscopic Thyroidectomy) was developed based on the Chinese version of the Thyroid Cancer-Specific Health-Related Quality of Life Questionnaire (THYCA-QoL)^9^. Given the unique anatomical considerations of TOETVA, in-depth interviews were individually conducted with patients, surgeons, and rehabilitation physicians. By amalgamating their perspectives, we incorporated additional items related to swallowing, sucking, occlusion, chewing, gum and dental function, and lip movement from the International Coach Federation (ICF) Core Suite of Clinical Practice (https://www.icf-core-sets.org/).

The questionnaire was divided into eight dimensions and 30 entries, including life assessment, own health status, interpersonal relationship, mucosal and muscular tissue damage, periodontal tissue damage, functional damage, pain, and functional damage of daily behavior. A Likert 5-point scale was used for scoring. For questions 1–11, the responses were valued as follows: "very satisfied" = 5, "satisfied" = 4, "average" = 3, "dissatisfied" = 2, and "very dissatisfied" = 1. For questions 12–30, the responses were scored in reverse: "very inconsistent" = 5, "not very consistent" = 4, "average" = 3, "more consistent" = 2, and "very consistent" = 1. This reverse scoring was used because higher scores indicate more difficulties in functional recovery, thus suggesting a slower recovery of quality of life.

#### Questionnaire revision

Using the Delphi expert consultation method, a total of two rounds of comments from the same experts for one month were collected for the structure of THYCA-QoL-TOETVA30 and for each entry within the eight dimensions, and revisions were made until final agreement was reached.

#### Expert correspondence

##### First round of expert correspondence

To ensure the accuracy of the consultation results and construct a questionnaire with good reliability, a total of 10 health care professionals with extensive clinical practice experience in thyroid surgery, postoperative rehabilitation, patient involvement, and medical quality management were selected as consultation experts in this study.

The expert consultation questionnaire consisted of three parts. The first part contains the consultation-related contents and instructions for filling out the questionnaire; the second part contains basic information about the consultation experts; and the last part contains the contents of each entry in the preliminary questionnaire. The experts were asked to determine whether each entry met the corresponding dimension and give the corresponding score on a 5-point scale, and at the same time to make comments or suggestions for modification, and to specify whether the entry should be retained or deleted. The correspondence questionnaire was collected within one week. The team members sorted, analyzed and discussed the collected questionnaires, adjusted and modified the entries accordingly, and formed the second round of expert questionnaires.

##### Second round of expert correspondence

After a 2-week interval, a second round of correspondence questionnaires were sent to each expert, and the process was the same as the first round. The questionnaires were returned within one week, and the panelists again summarized and discussed the experts’ opinions to form a pre-survey questionnaire.

### Questionnaire pre-survey and formal survey

Questionnaires were generated on the Wenjuanxing (https://www.wjx.cn) platform. Pre-survey patients were interviewed about questionnaire content, acceptance, and language wording. After the pre-survey, there were no modifications in the presentation of the questionnaire entries to form the official questionnaire. After obtaining patients’ consent, the data were collected by two members of the research team who were trained to distribute, explain, and retrieve the data in a uniform manner online, based on the principle of voluntary participation and withdrawal of patients. The filling time of patients was 6months after surgery.

### Cervical spine mobility measurement (Fig. 1)

**Figure 1:**
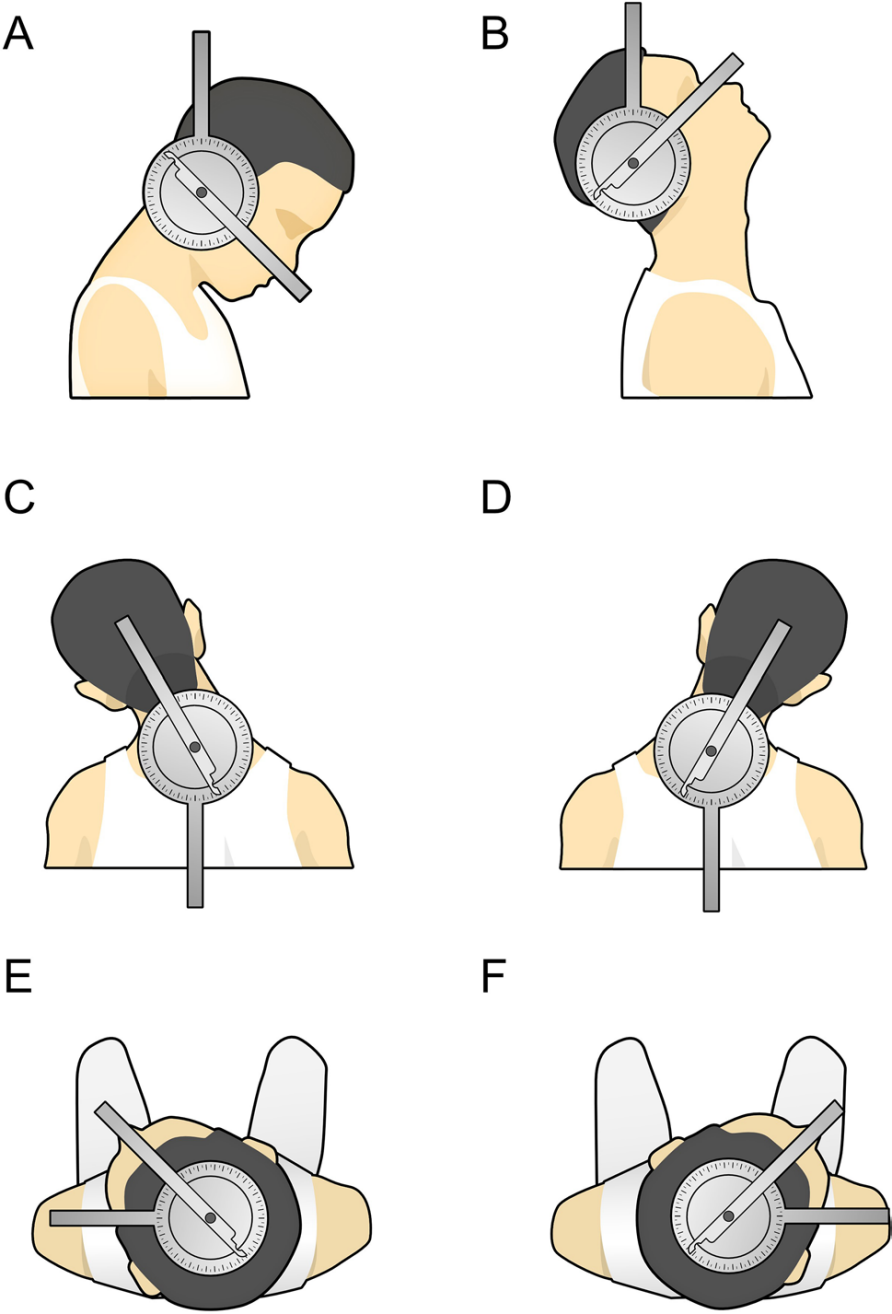
Cervical spine mobility measurement. (A) Flexion. (B) Extension. (C) Left lateral bending. (D) Right lateral bending. (E) Left axial rotation. (F) Right axial rotation.

Range of Motion (ROM) refers to the maximum arc that can be achieved when the joint moves, and is a measure of the amount of movement of a joint, and is one of the most basic elements of the limb movement function examination^10^. The Cervical Range of Motion (CROM), on the other hand, is an application of the ROM to the neck and is specifically designed to assess the mobility of the neck joints. For TOETVA patients, CROM is measured by thyroid surgeons at 1 and 3 months postoperatively to monitor the recovery progress of cervical function. For measurement, an 8-in. joint angle ruler was selected and the axis of the goniometer was aligned with the center of the joint’s axis of motion for measurement. To measure flexion and extension, the patient is asked to sit in the end position with the goniometer on the axis of the acromion, the fixed arm in the sagittal plane in line with the vertical line through the acromion, and the mobile arm in line with the line between the top of the head and the ear hole. The patient is asked to flex the neck so that the lower jaw is close to the chest or tilt the head so that the dorsal side of the head is close to the thoracic spine. To measure left and right axial rotation, the patient is asked to take a sitting position, the goniometer is centered on the top of the head, the fixed arm is aligned with the sagittal axis through the top of the head, and the mobile arm is aligned with the line between the bridge of the nose and the occipital ridge, the patient’s head is in a neutral position and then rotated from right to left, usually 0°–70° for left and right rotation. To measure left lateral bending and right lateral bending, the patient is asked to take a sitting position, and the goniometer is centered on the axis of the spinous process of the 7th cervical vertebra, with the fixed arm in line with the line from the 5th cervical vertebra to the spinous process of the 7th cervical vertebra, and the mobile arm in line with the line from the occipital ridge to the spinous process of the 7th cervical vertebra, and the patient is asked to flex the neck laterally so that the ear moves toward the shoulder. Measurements were made during outpatient follow-up visits at one and three months after surgery.

### Statistical analysis

Cronbach’s alpha coefficient was used to reflect the internal consistency reliability of the scale and the retest reliability of repeated measures. The structural validity of the scales was analyzed using exploratory factor analysis, and the Kaiser–Meyer–Olkin (KMO) values and Bartlett’s test of sphericity (BTS) were calculated to determine whether factor analysis was appropriate using Exploratory Factor Analysis (EFA). Principal component analysis was used to extract factors. The dimensionality was divided using the rotated component matrix. The experimental and control groups were subjected to Propensity Score Matching (PSM), and the caliper value was set to 0.03. The matched data of the two groups were tested for normality, and if they conformed to the normal distribution the independent samples t-test was used, and if they did not, the rank sum test was used. The cervical spine mobility of the experimental and control groups were grouped and then subjected to repeated measures ANOVA, and if the Mauchly’s test of sphericity was greater than 0.05 the within-subjects effect test was used as the result for comparison between groups, and if it was less than 0.05 the multivariate test was used as the result. SPSS 25.0 software was used to perform statistical processing of the data, with measures expressed as (SD) and counts expressed as cases (%), and in addition, t-tests were used to compare patients’ baseline data. The test level for all hypothesis tests was set at α = 0.05.

### Ethics approval and consent to participate

The study was approved by the Medical Ethics Committee of Hunan Cancer Hospital and the subjects gave their informed consent for participation.

## Results

### Delphi expert consultation results

The expert authority degree of the Delphi expert opinion is tested by the authoritative coefficient of experts, which is generally considered as greater than 0.7 to indicate an acceptable degree of authority. The authoritative coefficient of experts is 0.93 in the first round of the Delphi survey in this questionnaire and 0.95 in the second round, indicating a good degree of expert authority (Table 1).

**Table 1:** Authoritative coefficient of experts.

Round	N	Cs	Ca	Cr
Frist round	10	0.94	0.92	0.93
Second round	10	0.94	0.96	0.95

N: Sample number.

Cs: Score of their acquaintance with the questions.

Ca: Score of judgment basis.

Cr: Authoritative coefficient.

Coordination is tested by the Kendall coordination coefficient. Higher values indicate increased coordination. Statistics showed that the expert coordination of this questionnaire was 0.771 and 0.762 (Table 2).

**Table 2:** Coordination of experts’ opinions.

Round	Kendall’s W	c2	P value
First round	0.771	246.877	< 0.001
Second round	0.762	220.969	< 0.001

### Reliability analysis

The reliability of this questionnaire was judged by using Cronbach’s α coefficient to determine the reliability of the index, and a Cronbach’s α coefficient above 0.7 is generally considered to indicate good reliability of the questionnaire. The Cronbach′s α coefficient of this questionnaire was 0.717, and the repeat survey after 2–3 weeks showed that the retest reliability of the scale was 0.785, which was above 0.7, and proved that this questionnaire had high internal consistency and good reliability.

### Validity analysis

#### Content validity

The item-level CVI (I-CVI) ranged from 0.830 to 1.000, and the scale-level CVI (S-CVI) was 0.95. The content validity of this questionnaire was good.

#### Structural validity

Exploratory factor analysis was performed by applying SPSS 25.0. The results showed that the KMO value was 0.749 and the Bartlett’s spherical test χ^2^ was 2697.412, p < 0.01. We eliminated the questions "Do you have food residue left in your mouth when you eat?" and "How would you rate your quality of life?" from the initial questionnaire because the results of rotated component matrix were less than 0.5. The scale was extracted by exploratory factor analysis with 9 common factors, and the cumulative variance contribution rate was 66.711%. This suggests that the information covered by the extracted 9 common factors can reflect the thyroid cancer-specific quality of life issues more comprehensively, and has good structural validity. The question "Do you have respiratory impairment?” needs to be listed as a separate dimension and has components related to the upper respiratory tract, so it was deleted.

### Questionnaire analysis

**Figure 2:**
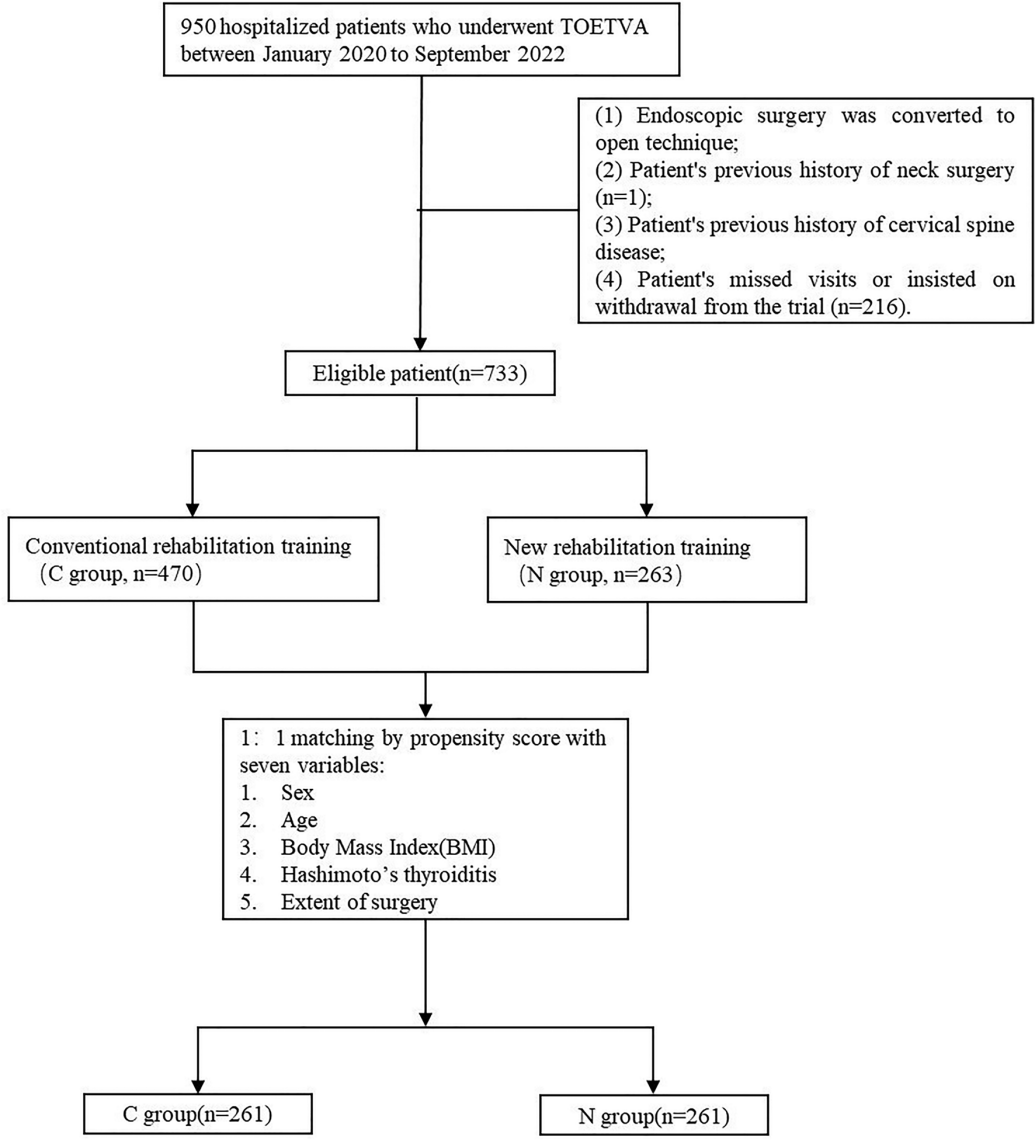
Flow chart of inclusion, exclusion, and propensity score matching.

A total of 733 questionnaires were collected during the study period, 263 in the experimental group and 470 in the control group. After matching scores by PSM, 522 patients (group N n = 261 and group C n = 261) remained in the study population (Fig. 2), and both groups were well balanced with respect to the five covariates. Patient demographics and clinical characteristics are summarized in Table 3. After PSM, there were no significant differences between the two groups in terms of gender, age, BMI, comorbid thyroiditis, and extent of surgery (Table 3).

**Table 3:** Demographics and clinical characteristics from the T and N groups before and after PSM.

Variables	Before PSM			After PSM		
	C group (n = 470)	N group (n = 263)	*P* value	C group (n = 261)	N group (n = 261)	*P* value
Sex, NO. (%)			0.009			1.000
Male	49 (10)	46 (17)		43(16)	43(16)	
Female	421 (90)	217 (83)		218 (84)	218 (84)	
Age, mean (SD), years	40.10 ± 9.56	40.56 ± 11.04	0.565	41.28 ± 9.54	40.40 ± 10.90	0.328
BMI, mean (SD)	22.88 ± 3.08	23.01 ± 3.51	0.600	23.14 ± 3.24	22.99 ± 3.49	0.622
Hashimoto’s thyroiditis, NO. (%)			0.116			0.471
Yes	160(34)	105(40)		95(36)	103(39)	
No	310(66)	158 (60)		166 (64)	158 (61)	
Extent of surgery, NO. (%)			0.124			0.541
Hemithyroidectomy with CND	253 (54)	126 (48)		133(51)	126 (48)	
Bilateral thyroidectomy with CND	217(46)	137 (52)		128 (49)	135 (52)	

We assigned a reverse value to questions 12–30. The independent samples t-test showed that t(df) = − 2.228 (519.712), *p* = 0.026, indicating that the difference between the N group score (124.55 ± 8.171) and the C group score (122.94 ± 8.366) was statistically significant and that the N group had better recovery and quality of life than the C group (Table 4).

**Table 4:** Two-sample t-test of C group and N group.

	N	M	SD	t(df)	*P* value
C group	261	122.94	8.366	−	0.026
N group	261	124.55	8.171	2.228 (519.712)	

### Cervical spine mobility analysis

CROM measurements of flexion, extension, left lateral bending, right lateral bending, left axial rotation, and right axial rotation in the N group and C group were not significantly different preoperatively (*p* > 0.05).

There were significant differences in CROM measurements for flexion, extension, left lateral bending, right lateral bending, left axial rotation, and right axial rotation between the two groups at one month and three months postoperatively (*p* < 0.001) (Table 5). Repeated measures ANOVA on the above data showed Mauchly W values of 0.807, 0.849, 0.883, 0.935, 0.820, and 0.843 (*p* < 0.001), respectively, which did not conform to the Mauchly’s test of sphericity (Table 6). The results of multivariate tests showed significant interaction effects between measurement time points and groups, with F values of 869.328, 1545.869, 278.792, 211.035, 168.069, and 167.008, respectively (*p* value all of these < 0.001), and bias η^2^ of 0.770, 0.983, 0.518, 0.449, 0.393, and 0.392, with significant changes in CROM measurements of cervical mobility over time in both the N group and C group (Table 7). The results of simple effects analysis showed that there was no difference in CROM measurements of flexion, extension, left lateral bending, right lateral bending, left axial rotation, and right axial rotation between the N group and C group patients at admission. In comparison to Group C, Group N exhibits significantly superior postoperative rehabilitation outcomes. At 1 month after surgery, the increases in flexion, extension, left lateral bending, right lateral bending, left axial rotation and right axial rotation were 1.762°, 4.720°, 3.912° 4.061°, and 5.180° and 5.199°, respectively, surpassing those of Group C. The 3-month postoperative measurements reveal similar improvements, with increments of 2.866°, 2.904°, 3.927°, 3.330°, 4.395°, and 3.992°. These differences were all statistically significant (*p* < 0.001) (Table 8).

**Table 5:** Univariate analysis of CROM over 3 months.

Measurement	Preoperative	*P* value	Postoperative 1 month	*P* value	Postoperative 3 months	*P* value
Flexion, mean (SD), °		0.097		< 0.001		< 0.001
C group	44.79 ± 2.768		37.54 ± 2.997		40.53 ± 2.817	
N group	44.38 ± 2.817		39.31 ± 2.980		43.40 ± 2.704	
Extension, mean (SD), °		0.966		< 0.001		< 0.001
C group	45.99 ± 2.106		31.10 ± 2.437		36.24 ± 2.300	
N group	46.00 ± 2.048		35.82 ± 2.271		39.14 ± 2.390	
Left lateral bending, mean (SD), °		0.698		< 0.001		< 0.001
C group	46.63 ± 1.354		36.00 ± 2.829		41.18 ± 2.671	
N group	46.58 ± 1.352		39.92 ± 2.392		45.11 ± 1.448	
Right lateral bending, mean (SD), °		0.115		< 0.001		< 0.001
C group	46.89 ± 1.368		36.10 ± 2.497		41.89 ± 2.772	
N group	46.69 ± 1.512		40.16 ± 2.731		45.21 ± 1.787	
Left axial rotation, mean (SD), °		0.265		< 0.001		< 0.001
C group	64.79 ± 3.127		55.11 ± 2.209		59.18 ± 2.412	
N group	65.08 ± 2.833		60.29 ± 2.176		63.57 ± 2.775	
Right axial rotation, mean (SD), °		0.191		< 0.001		< 0.001
C group	65.29 ± 3.234		55.31 ± 2.540		59.81 ± 2.633	
N group	65.19 ± 3.039		60.51 ± 2.491		63.80 ± 2.794

**Table 6:** Mauchly’s test of sphericity for CROM.

Within Subject Effect	Measurement	Mauchly’s W	Approx. Chi-Square	df	*P* value	Greenhouse–Geisser	Huynh–Feldt	Lower-bound
Time point	Flexion	0.807	111.167	2	< 0.001	0.838	0.842	0.500
	Extension	0.849	84.876	2	< 0.001	0.869	0.873	0.500
	Left lateral bending	0.883	64.378	2	< 0.001	0.896	0.900	0.500
	Right lateral bending	0.935	35.12	2	< 0.001	0.939	0.944	0.500
	Left axial rotation	0.820	103.284	2	< 0.001	0.847	0.851	0.500
	Right axial rotation	0.843	88.934	2	< 0.001	0.864	0.868	0.500

**Table 7:** Repeated measures F-test for CROM.

Measurement	F	P value	Partial η^2^
Flexion	869.328	< 0.001	0.770
Extension	1545.869	< 0.001	0.983
Left lateral bending	278.792	< 0.001	0.518
Right lateral bending	211.035	< 0.001	0.449
Left axial rotation	168.059	< 0.001	0.393
Right axial rotation	167.008	< 0.001	0.392

**Table 8:** Simple effect analysis of CROM over 3 months.

Measurement	Time point	Mean Difference (N group- C group, °)	Std. error	p value
Flexion	Preoperative	− 0.067	0.244	0.097
	Postoperative 1 month	1.762	0.262	< 0.001
	Postoperative 3 months	2.866	0.242	< 0.001
Extension	Preoperative	0.008	0.182	0.966
	Postoperative 1 month	4.720	0.206	< 0.001
	Postoperative 3 months	2.904	0.205	< 0.001
Left lateral bending	Preoperative	− 0.046	0.118	0.698
	Postoperative 1 month	3.912	0.229	< 0.001
	Postoperative 3 months	3.927	0.188	< 0.001
Right lateral bending	Preoperative	− 0.199	0.126	0.115
	Postoperative 1 month	4.061	0.229	< 0.001
	Postoperative 3 months	3.330	0.204	< 0.001
Left axial rotation	Preoperative	0.291	0.261	0.265
	Postoperative 1 month	5.180	0.192	< 0.001
	Postoperative 3 months	4.395	0.228	< 0.001
Right axial rotation	Preoperative	− 0.100	0.275	0.717
	Postoperative 1 month	5.199	0.220	< 0.001
	Postoperative 3 months	3.992	0.238	< 0.001

## Discussion

While PTC exhibits a high incidence rate, its prognosis remains favorable^11^. In recent years, there has been an increasing focus on the postoperative QOL among thyroid cancer patients. In 1997, DOW et al*.* ^12^ introduced the City of Hope Quality of Life Questionnaire—The QOL Thyroid Scale (TCC HQ), marking the initiation of QOL assessments specific to thyroid cancer. Subsequently, other questionnaires such as the EORTC QLQ-THY34 and THYCA-QoL have been developed^13–15^. Despite the availability of numerous questionnaires for assessing the QOL in thyroid cancer patients, it is worth noting that generic tools may not effectively address issues specific to certain populations.

In recent years, TOETVA has gained increasing popularity among both patients and physicians as the sole procedure aligning with the principles of Natural Orifice Translumenal Endoscopic Surgery (NOTES) ^1,16^. However, TOETVA employs a subcutaneous access through the oral vestibule, necessitating the separation of structures above the broad cervical muscle in the anterior cervical region ^17^. This technique carries the potential for a prolonged pulling sensation under the jaw, attributed to fibrosis, flap perforation, and skin burns resulting from electrotherapy^18,19^. These factors may impact the postoperative recovery of patients.

Improving the quality of life of thyroid cancer patients is one of the objectives of cancer treatment^9,20^. However, there is no questionnaire specifically designed to assess the quality of life of TOETVA patients after surgery, and the THYCA-QoL questions for the oral cavity and anterior cervical region are limited to "Did the scar in your neck bother you?", and there are no specific questions about mucosal and muscular tissue damage or periodontal tissue damage. Taking the aforementioned concerns into account, extensive interviews were undertaken involving patients, surgeons, and rehabilitation physicians. Subsequently, we devised a postoperative quality of life questionnaire tailored for TOETVA patients. This questionnaire was constructed by adapting an existing tool designed for evaluating postoperative quality of life in thyroid cancer, encompassing 30 items across eight dimensions. The aim was to comprehensively assess the anatomical impact of the TOETVA procedure on patients’ postoperative quality of life.

A study ^21^ established that I-CVI ≥ 0.78 and S-CVI ≥ 0.9 were indicative of robust content validity. The final I-CVI of this study ranged from 0.83 to 1.000 and the S-CVI was 0.95, indicating that this scale has good content validity. Structural validity analysis was conducted by exploratory factor analysis. The results showed that the KMO was 0.873, indicating a good relationship between the variables, and the cumulative variance contribution was 60.549%, which had good structural validity. The Cronbach’s α coefficient of this study scale was 0.829, and the split-half reliability coefficient was 0.718. Compared with various Chinese versions of the thyroid cancer quality of life scale^22,23^, this scale has good accuracy, stability and internal consistency. Through the reliability analysis of this questionnaire we found that this questionnaire is an effective representation of the postoperative quality of life of TOETVA patients. There is a clearer orientation for the study of postoperative quality of life in this group.

As a recently introduced endoscopic thyroidectomy, the TOETVA induces postoperative pain at different levels and sites compared to conventional methods^1^. It is noteworthy that, despite TOETVA involving fewer dissected tissue planes and causing less pain than other endoscopic thyroidectomy^24^, pain can still significantly impact the postoperative quality of life for patients^25,26^. Additionally, it has been recognized that targeted and effective rehabilitation interventions during the treatment phase can substantially enhance the quality of life for patients^4^. Given the absence of rehabilitation training for TOETVA patients, this study collaborated with the Department of Rehabilitation Medicine to formulate a novel rehabilitation program focusing on the oral cavity, anterior cervical fascia, and superficial neck and shoulder fascia pertinent to the anatomy of TOETVA. Encouragingly, a survey using our newly designed questionnaire revealed that the overall postoperative quality of life in the group undergoing the novel rehabilitation program surpassed that of the control group. This not only highlights the efficacy of our innovative rehabilitation approach but also underscores the validity of the newly developed questionnaire.

The positive correlation observed between the enhanced postoperative quality of life and the novel rehabilitation method suggests that tailoring rehabilitation strategies to the unique challenges posed by the TOETVA contributes significantly to patients’ overall well-being. The targeted exercises and interventions designed for the oral cavity, anterior cervical fascia, and superficial neck and shoulder fascia appear to address specific issues related to TOETVA, ultimately leading to improved postoperative outcomes and a higher quality of life for the patients.

CROM is a frequently used evaluation index in rehabilitation medicine, serving as a common standard for clinical examination of the cervical spine^27,28^. According to the literature, thyroid cancer, regardless of the procedure, may lead to swelling of the peri-cervical tissues and further restriction of neck movement due to prolonged surgical trauma and scar contracture of the incision^29^. In particular, TOETVA requires the establishment of a subcutaneous access through the oral vestibule, with an opening above the inferior labial tether and blunt separation to the superior border of the thyroid cartilage and the anterior border of the sternocleidomastoid muscle on both sides^30–32^. This predisposes the broad cervical and subglottis muscle groups to injury from the establishment of surgical access. Unlike the limitation of neck mobility due to organic lesions of the cervical spine, scarring of the submandibular musculature after TOETVA affects the pulling of the muscle itself, thus causing the same limitation of neck mobility^18^. The significance of the novel rehabilitation method employed in this study becomes particularly evident when considering the unique challenges posed by the TOETVA procedure and its impact on neck mobility. The utilization of CROM as an objective criterion for evaluating neck mobility in post-TOETVA patients provided a standardized and measurable parameter, allowing for a comprehensive assessment of the effectiveness of the rehabilitation intervention.

The results obtained from CROM measurements at different time points—preoperative, one month postoperative, and three months postoperative—highlighted the positive influence of the new rehabilitation training method. The significant improvement observed in neck mobility among patients undergoing the novel rehabilitation program implies a targeted and beneficial impact on the anatomical structures affected by the TOETVA procedure. This underscores the importance of tailoring rehabilitation strategies to the unique anatomical considerations of emerging surgical techniques. By specifically addressing the challenges associated with TOETVA, the rehabilitation program not only demonstrates its efficacy in promoting neck mobility but also emphasizes the potential for improving overall postoperative quality of life for patients. Enhanced neck mobility is crucial for daily activities and quality of life, and the positive outcomes observed in this study suggest that a specialized rehabilitation approach can contribute significantly to the recovery and well-being of patients undergoing TOETVA.

However, it is crucial to note that we produced instructional videos for the new rehabilitation program, distributing them to each patient in the experimental group. In contrast, for the control group, we presented only images of the rehabilitation exercises. This discrepancy in the mode of delivery may potentially result in varying levels of emphasis on postoperative rehabilitation among patients. The visual component of instructional videos could contribute to a more comprehensive understanding and engagement with the rehabilitation exercises, potentially influencing the effectiveness of the rehabilitation intervention. Therefore, while interpreting the positive outcomes observed in the experimental group, it is essential to consider the potential impact of the instructional method on patients’ commitment to and compliance with the rehabilitation program. Further investigations may be warranted to explore the role of instructional modalities in postoperative rehabilitation adherence and outcomes.

Our study still has some limitations. Firstly, this is a retrospective study, and some of the data were based on patient recall, which may be subject to recall bias. Next, the fact that only one questionnaire collection was performed is one of the limitations of this study. In addition, even when PSM score matching was used for the experimental and control groups, patient selection bias and confounding differences remained between the two groups. Finally, there may be measurement bias in the manual collection of CROM data in the outpatient clinic, which failed to perform accurate measurements due to the use of a single instrument.

## Conclusion

In conclusion, THYCA-QoL-TOETVA provides a more appropriate and valid assessment tool for evaluating the postoperative quality of life of TOETVA patients. The results of the THYCA-QoL-TOETVA and the cohort-controlled study we prepared suggest that the new rehabilitation training is effective for postoperative rehabilitation of TOETVA patients. The results of our study are encouraging in that postoperative rehabilitation training no longer focus only on the rehabilitation of the surgical area alone, but the inclusion of consideration and planning of the surgical access can improve the rationality and effectiveness of postoperative rehabilitation training. In addition, in the future, we will increase questionnaire delivery and precise questionnaire collection time to reduce errors due to time and frequency. And we will apply more accurate instruments as the standard when possible to reduce the measurement error of CROM.

## Contributions to the literature

Development of the THYCA-QoL-TOETVA questionnaire, offering a novel and specialized assessment tool tailored for evaluating the postoperative quality of life of TOETVA patients. Rehabilitation Training Advancement: Introduction of a new and effective rehabilitation training modality for TOETVA patients, addressing the unique challenges posed by the surgical approach and significantly improving cervical range of motion. Addressing Literature Gap: Filling a gap in the literature by focusing on the quality of life and rehabilitation of patients undergoing TOETVA, an area that has been relatively underexplored in existing research.

## Clinical implications

Practical implications for postoperative care, emphasizing the importance of considering surgical access in rehabilitation planning, thereby enhancing the rationality and effectiveness of postoperative rehabilitation training.

### Supplementary Information

Supplementary Information.

Supplementary Video 1.

## Data Availability

The datasets used and/or analysed during the current study are available from the corresponding author on reasonable request.

## Abbreviations

TOETVA: Transoral endoscopic vestibular thyroidectomy
PSM: Propensity score matching
BMI: Body mass index
ICF: International Coaching Federation
CROM: Cervical range of motion
ROM: Range of Motion
KMO: Kaiser-Meyer-Olkin
BTS: Bartlett’s test of sphericity
EFA: Exploratory Factor Analysis
NOTES: Natural Orifice Translumenal Endoscopic Surgery
TCC HQ: City of Hope Quality of Life Questionnaire-The QOL Thyroid Scale
EORTC: European Organization for Research and Treatment
EORTC QLQ-THY34: EORTC Quality of Life Module for Thyroid Cancer
EORTC QLQ-C30: European Organization for Research and Treatment of Cancer Quality of Life Questionnaire-Core30
THYCA-QoL: Thyroid Cancer Specific Health-related Quality of Life Questionnaire

## References

[1] Anuwong A, Ketwong K, Jitpratoom P, Sasanakietkul T, Duh Q-Y (2018). Safety and outcomes of the transoral endoscopic thyroidectomy vestibular approach. JAMA Surg..

[2] Shaha AR (2021). Thyroid surgery—Does the scar matter?. Arch. Endocrinol. Metab..

[3] Nitta K, Ishikawa N, Kawaguchi M, Ooi A, Watanabe G (2015). Thyroidectomy using pure natural orifice transluminal endoscopic surgery in a porcine model. Artif. Organs..

[4] Lewthwaite R, Winstein CJ, Lane CJ, Blanton S, Wagenheim BR, Nelsen MA (2018). Accelerating stroke recovery: Body structures and functions, activities, participation, and quality of life outcomes from a large rehabilitation trial. Neurorehabil. Neural. Repair..

[5] Thorsen RT, Døssing H, Bonnema SJ, Brix TH, Godballe C, Sorensen JR (2022). The impact of post-thyroidectomy neck stretching exercises on neck discomfort, pressure symptoms, voice and quality of life: A randomized controlled trial. World J. Surg..

[6] Husson O, Haak HR, Mols F, Nieuwenhuijzen GA, Nieuwlaat W-A, Reemst PH (2013). Development of a disease-specific health-related quality of life questionnaire (THYCA-QoL) for thyroid cancer survivors. Acta Oncol..

[7] von Elm E, Altman DG, Egger M, Pocock SJ, Gøtzsche PC, Vandenbroucke JP (2007). The Strengthening the Reporting of Observational Studies in Epidemiology (STROBE) statement: Guidelines for reporting observational studies. Lancet..

[8] Takamura Y, Miyauchi A, Tomoda C, Uruno T, Ito Y, Miya A (2005). Stretching exercises to reduce symptoms of postoperative neck discomfort after thyroid surgery: Prospective randomized study. World J. Surg..

[9] Jie L, Jing G, Yuan T, Chenxi W, Xiaolin J, Qin L (2019). Reliability and validity of Chinese version of Thyroid Cancer-specific Quality of Life (THYCA-QoL) questionnaire. Tumor..

[10] Dengkun N, Xiaolin H. Practice of Rehabilitation Medicine2009.

[11] Siegel RL, Miller KD, Wagle NS, Jemal A (2023). Cancer statistics, 2023. CA Cancer J. Clin..

[12] Dow KH, Ferrell BR, Anello C (1997). Quality-of-life changes in patients with thyroid cancer after withdrawal of thyroid hormone therapy. Thyroid..

[13] Singer S, Husson O, Tomaszewska IM, Locati LD, Kiyota N, Scheidemann-Wesp U (2016). Quality-of-life priorities in patients with thyroid cancer: A multinational European Organisation for Research and Treatment of Cancer Phase I Study. Thyroid..

[14] Aaronson NK, Ahmedzai S, Bergman B, Bullinger M, Cull A, Duez NJ (1993). The European Organization for Research and Treatment of Cancer QLQ-C30: A quality-of-life instrument for use in international clinical trials in oncology. J. Natl. Cancer Inst..

[15] Singer S, Jordan S, Locati LD, Pinto M, Tomaszewska IM, Araújo C (2017). The EORTC module for quality of life in patients with thyroid cancer: Phase III. Endocr. Relat. Cancer..

[16] Russell JO, Razavi CR, Shaear M, Chen LW, Lee AH, Ranganath R (2019). Transoral vestibular thyroidectomy: Current state of affairs and considerations for the future. J. Clin. Endocrinol. Metab..

[17] Liang Y, Zeyang L, Xiaowei P, Zan L, Bo Z, Chunliu L (2020). Indications and contraindications for transoral endoscopic thyroidectomy by vestibular approach. Chin. J. Otorhinolaryngol. Head Neck Surg..

[18] Bakkar S, Al Hyari M, Naghawi M, Corsini C, Miccoli P (2018). Transoral thyroidectomy: A viable surgical option with unprecedented complications—A case series. J. Endocrinol. Invest..

[19] Benhidjeb T, Witzel K, Stark M, Schulte Am Esch J (2019). Transoral thyroidectomy: New method with new complications’ spectrum. J. Endocrinol. Invest..

[20] Zhao, H., Jin, H., Xian, J., Zhang, Z., Shi, J., Bai, X. Effect of Ketogenic diets on body composition and metabolic parameters of cancer patients: A systematic review and meta-analysis. *Nutrients*. **14**(19) (2022). 10.3390/nu14194192 PMC9570668 36235844

[21] Jingcheng S, Xiankun M, Zhenqiu S (2012). Content validity index in scale development. J. Central S. Univ. (Med. Sci)..

[22] Xin W, Jie G, Min Z, Juan L, Li Z, Shulang W (2023). Chinese version of the European organisation for research and treatment of cancer quality of life module for thyroid cancer 34 and its reliability and validity. J. Nurses Training..

[23] Xia X, Xujuan X, Zhifeng G, Yi S, Qing D, Jing Z (2017). Reliability and validity of the Chinese version of short version of thyroid—Specific patient reported outcome. Chin. General Practice..

[24] Kim WW, Park CS, Lee J, Jung JH, Park HY, Tufano RP (2020). Real scarless transoral robotic thyroidectomy using three ports without axillary incision. J. Laparoendosc. Adv. Surg. Tech. A..

[25] Liu SS, Wu CL (2007). Effect of postoperative analgesia on major postoperative complications: A systematic update of the evidence. Anesth. Analg..

[26] Lahtinen P, Kokki H, Hynynen M (2006). Pain after cardiac surgery: A prospective cohort study of 1-year incidence and intensity. Anesthesiology..

[27] Schneider KJ, Meeuwisse WH, Nettel-Aguirre A, Barlow K, Boyd L, Kang J (2014). Cervicovestibular rehabilitation in sport-related concussion: A randomised controlled trial. Br. J. Sports Med..

[28] Grider-Potter N, Nalley TK, Thompson NE, Goto R, Nakano Y (2020). Influences of passive intervertebral range of motion on cervical vertebral form. Am. J. Phys. Anthropol..

[29] Jing H, Xinya Z, Guanmian L, Chun H (2016). Collective exercise neck and shoulder operation in the application of the thyroid carcinoma postoperative early functional rehabilitation. Chin. J. Modern Nursing..

[30] Chai YJ, Chung JK, Anuwong A, Dionigi G, Kim HY, Hwang K-T (2017). Transoral endoscopic thyroidectomy for papillary thyroid microcarcinoma: Initial experience of a single surgeon. Ann. Surg. Treat. Res..

[31] Wang Y, Yu X, Wang P, Miao C, Xie Q, Yan H (2016). Implementation of intraoperative neuromonitoring for transoral endoscopic thyroid surgery: A preliminary report. J. Laparoendosc. Adv. Surg. Tech. A..

[32] Jongekkasit I, Jitpratoom P, Sasanakietkul T, Anuwong A (2019). Transoral endoscopic thyroidectomy for thyroid cancer. Endocrinol. Metab. Clin. N. Am..

