# Supplementary material for: Postoperative rehabilitation and quality of life evaluation for transoral endoscopic thyroidectomy vestibular approach
Source: Sci Rep. 2024 Jun 26;14:14747. doi: 10.1038/s41598-024-65589-x (PMC11208515; doi:10.1038/s41598-024-65589-x)
Supplement: Supplementary file 1 — Supplementary Information. [file 41598_2024_65589_MOESM1_ESM.docx]

**生活评估 (Life assessment)**

1. 您觉得自己的生活有意义吗？ (Do you feel that your life is meaningful?)
2. 您觉得生活有乐趣吗？ (Do you find life to be enjoyable?)
3. 日常生活中您感觉安全吗？ (Do you feel safe in your daily life?)
4. 您能集中注意力吗？ (Are you able to concentrate?)
5. 您的生活环境对健康好吗？ (Is your living environment good for your health?)

**自身健康状况 (Own health status)**

1. 您对自己健康状况满意吗？ (Are you satisfied with your health condition?)
2. 您对自己的睡眠情况满意吗? (Are you satisfied with your sleep?)
3. 您对保持日常生活的医学治疗的需求程度有多大？ (How great is your need for medical treatment to maintain daily life?)

**人际关系 (Interpersonal relationship)**

1. 您对自己直系亲属之间（父母/子女/兄弟姐妹）的关系满意吗? (Are you satisfied with the relationship between your immediate family members (parents/children/siblings)?)
2. 您对自己旁系亲属之间（叔伯/姨妈/姑妈/表兄弟姐妹）的关系满意吗? (Are you satisfied with the relationship between your extended family members (uncles/aunts/cousins)?)
3. 您对自己从朋友那里得到的支持满意吗? (Are you satisfied with the support you receive from friends?)

**粘膜和肌肉组织损伤 (Mucosal and muscular tissue damage)**

1. 您的下颌至颈部肌肉有损伤吗？ (Do you have any injuries to the muscles from your jaw to your neck?)
2. 您的头颈部关节活动能力有损伤吗？如低头/抬头/左右侧屈/左右旋转 (Is there any impairment in your head and neck joint mobility? Such as bending down/looking up/side bending/rotation left and right)
3. 您的口腔有损伤吗？ (Do you have any oral injuries?)
4. 您的下颌至颈部皮肤有损伤吗？ (Do you have any skin injuries from your jaw to your neck?)
5. 您的嘴唇有损伤吗？ (Do you have any lip injuries?)

**牙周组织损伤 (Periodontal tissue damage)**

1. 您的牙齿有损伤吗？ (Do you have any tooth injuries?)
2. 您的牙龈有损伤吗？ (Do you have any gum injuries?)
3. 您用前牙咬碎、磨碎或撕开食物的功能有损伤吗？ (Is there any impairment in your ability to crush, grind, or tear food with your front teeth?)
4. 您用后牙（如磨牙）碾、磨和咀嚼食物的功能有损伤吗？ (Is there any impairment in your ability to grind and chew food with your back teeth (such as molars)?)

**功能损伤 (Functional damage of daily behavior)**

1. 您的发音、声调、响度和其他音质功能有损伤吗？如失声、发声困难、声嘶、鼻音过重、鼻音过轻的损伤 (Do you have impairments in articulation, voice pitch, loudness and other phonological functions? Impairments such as loss of voice, vocal difficulties, hoarseness, hypernasality, hyponasality)
2. 您通过正常方式吃/喝东西的能力有损伤吗（将食物送进嘴中并按有教养的方式吃喝以及使用吸管或饮用像水龙头或泉眼的流水） (Is there any impairment in your ability to eat/drink normally (conveying food into the mouth and eating/drinking in a mannered way as well as using a straw or drinking from a faucet or spring)?)
3. 您咀嚼、口中控制、吞咽食物的能力有损伤吗？ (Is there any impairment in your ability to chew, control food in your mouth, and swallow?)
4. 您的吸允功能（通过颊、唇和舌的运动产生吸力将食物纳入口中的功能）有损伤吗？ (Is there any impairment in your sucking function (the ability to draw food into the mouth through the movement of cheeks, lips, and tongue)?)

**疼痛损伤 (Pain damage of daily behavior)**

1. 您有全身性疼痛吗？包括刺痛，钝痛，灼痛等 (Do you experience generalized pain, including pricking, dull, burning, etc.?)
2. 您有局部性疼痛吗？包括皮肤疼痛、刺痛、灼痛、钝痛、牵拉痛吗？ (Do you experience localized pain, including skin pain, pricking, burning, dull, pulling pain?)
3. 您因躯体疼痛而妨碍您去做需要做的事感到有多烦恼？ (How troubled are you by physical pain that hinders you from doing what you need to do?)

**日常行为功能损伤 (Functional damage of daily behavior)**

1. 您的呼吸频率、节律、深呼吸能力有损伤吗？如呼吸暂停、通气过度、不规则呼吸、逆向呼吸、支气管痉挛和肺气肿的损伤？ (Is there any impairment in your breathing frequency, rhythm, and deep breathing capacity? Such as apnea, hyperventilation, irregular breathing, reverse breathing, bronchospasm, and emphysema?)
2. 您安排和完成日常事务的能力有损伤吗？ (Is there any impairment in your ability to organize and complete daily tasks?)
3. 您正常行动的能力有损伤吗? (Is there any impairment in your ability to move normally?)
